# Real-world treatment sequencing and survival in previously treated advanced renal cell carcinoma patients receiving nivolumab monotherapy: a UK retrospective cohort study

**DOI:** 10.1186/s12885-022-09694-y

**Published:** 2022-06-06

**Authors:** T. Waddell, K. Fife, R. Griffiths, A. Sharma, P. Dhokia, L. Groves, M. Hurst, C. Tsang, D. Sugrue, S. McKenna, J. Houghton, R. Carroll

**Affiliations:** 1 Gastro-Oesophageal and Renal Unit, The Christie NHS Foundation Trust, Manchester, UK; 2 Cambridge University NHS Foundation Trust, Cambridge, UK; 3 The Clatterbridge Cancer Centre, Birkenhead, UK; 4 Mount Vernon Cancer Centre, Northwood, UK; 5 Bristol Myers Squibb, Uxbridge, UK; 6 Health Economics & Outcomes Research Ltd, Cardiff, UK

**Keywords:** Nivolumab, Medical record review, Renal cell carcinoma, Survival, Real-world evidence

## Abstract

**Background:**

The CheckMate 025 trial established nivolumab monotherapy as one of the standards of care in previously treated advanced or metastatic renal cell carcinoma (aRCC). However, supporting real-world data is lacking. This study investigated characteristics, treatment sequences and clinical outcomes of patients who received nivolumab monotherapy for previously treated aRCC in the UK.

**Methods:**

This was a retrospective cohort study of aRCC patients treated with nivolumab at second line or later (2L +) at 4 UK oncology centres. Eligible patients commenced nivolumab (index date) between 01 March 2016 and 30 June 2018 (index period). Study data were extracted from medical records using an electronic case report form. Data cut-off (end of follow-up) was 31 May 2019.

**Results:**

In total, 151 patients were included with median follow-up of 15.2 months. Mean age was 66.9 years, male preponderance (72.2%), and mostly Eastern Cooperative Oncology Group performance status grade 0–1 (71.5%). Amongst 112 patients with a known International Metastatic RCC Database Consortium score, distribution between favourable, intermediate, and poor risk categories was 20.5%, 53.6%, and 25.9% respectively.

The majority of patients (*n* = 109; 72.2%) received nivolumab at 2L, and these patients had a median overall survival (OS) of 23.0 months [95% confidence interval: 17.2, not reached]. All patients who received nivolumab at 2L had received TKIs at 1L. Amongst the 42 patients (27.8%) who received nivolumab in third line or later (3L +) the median OS was 12.4 months [95% CI: 8.8, 23.2]. The most common reasons for nivolumab discontinuation were disease progression (2L: 61.2%; 3L: 68.8%) and adverse events (2L: 34.7%; 3L: 28.1%).

**Conclusion:**

This study provides real-world evidence on the characteristics, treatment sequences, and outcomes of aRCC patients who received 2L + nivolumab monotherapy in the UK. Nivolumab-specific survival outcomes were similar to those achieved in the CheckMate 025 trial.

## Background

Kidney cancer poses a significant burden to public health and clinical practice, with Europe having some of the highest incidence rates in the world [1]. In the United Kingdom (UK), kidney cancer is the 7th most common cancer, accounting for 4% of all new cancer cases and with approximately 13,100 new diagnoses per year, based on published data from 2015–2017 [2]. The incidence of kidney cancer is projected to rise by 26% between 2014 to 2035, to 32 cases per 100,000 people [2]. The most common type of kidney cancer is renal cell carcinoma, which account for approximately 80% of cases [3]. When diagnosed at its earliest stage (stage I), 96% of patients survive for a year or more, whereas in advanced or metastatic renal cell carcinoma (aRCC) only 39% of patients survive for a year or more [4]. In the UK, between 25 and 31% of patients diagnosed with kidney cancer have aRCC at diagnosis [5–8]. Additionally, amongst patients who have curative resection for early stage RCC (stages I and II), approximately half go on to develop advanced and/or metastatic disease at a later timepoint [6].

The treatment options for aRCC have expanded over the last decade with the introduction of targeted agents including tyrosine kinase inhibitors (TKIs) and inhibitors of vascular endothelial growth factor (VEGF) and mammalian target of rapamycin (mTOR) [9, 10]. More recently, the introduction of immune checkpoint inhibitors (ICIs) has significantly improved patient outcomes and has provided clinicians with greater choice and flexibility when selecting anti-cancer therapies [11]. Nivolumab is an ICI that blocks the interaction between programmed cell death protein-1 (PD‐1) expressed on T cells and its ligands programmed cell death protein ligand-1 (PD‐L1) and PD‐L2, expressed on antigen‐presenting cells and cancer cells [12, 13]. The efficacy and safety of nivolumab has been demonstrated in CheckMate 025 (NCT0166874), a phase III randomised controlled trial (RCT) of nivolumab monotherapy in patients with refractory aRCC [14]. Patients in the nivolumab monotherapy arm had significantly longer median overall survival (OS) and more favourable safety outcomes compared with patients who received the comparator, everolimus [15, 16]. In the UK, nivolumab was approved for previously treated advanced renal cell carcinoma by the National Institute for Health and Care Excellence in 2016 [17].

Due to the high selectivity of clinical trial populations and controlled study conditions, trial outcomes may not represent the wider aRCC population in routine clinical practice [18]. Supplementary real-world evidence (RWE) may support and complement the evidence generated through clinical trials by providing information on the characteristics of patients, treatment sequences, and outcomes that are more reflective of real-world clinical practice [18]. Therefore, the aim of this study was to generate RWE on the treatment sequences and clinical outcomes of patients with previously treated aRCC who received nivolumab monotherapy in the UK.

## Methods

### Study design

This was a multi-centre, retrospective cohort study of adult patients with previously treated aRCC who received nivolumab monotherapy in the UK. Data were collected from medical records at four specialist National Health Service (NHS) cancer treatment centres in England.

The study was approved by the Health Research Authority (Reference: 257,930). A minor amendment was submitted to the HRA and approved on 23 June 2020. Ethical approval was received from the Yorkshire & The Humber – Bradford Leeds Research Ethics Committee (Reference: 19/YH/0185).

### Study sample

Eligible patients were identified through screening of medical records at participating secondary care study centres. Patients were included in the study if they met all study eligibility criteria: ≥ 18 years at RCC diagnosis; had a primary diagnosis of aRCC; and received nivolumab monotherapy for aRCC as under licenced uses (second or subsequent lines of therapy[LOTs]) during the index period (01 March 2016 to 30 June 2018). Patients were excluded if they were diagnosed with malignant tumours other than RCC, had incomplete treatment information, had inadequate treatment follow-up (i.e., < 6 months post index date [date of nivolumab monotherapy initiation for aRCC]), received nivolumab at first line (1L), or received nivolumab within a clinical trial.

### Data collection

Data were sourced from the medical records of a sample of eligible patients at each study centre from the date of initiation of the first treatment for aRCC to the earliest of the following: most recent visit, death, or end of follow up (EOFU) on 31 May 2019. The study window was chosen to provide a contemporaneous dataset of patients with up to three years of follow up data. Clinical data were extracted from medical records at each study centre and collected for this study using a bespoke electronic case report form (eCRF) in Microsoft Excel. The study investigators (SIs) at each study centre were responsible for patient identification, eligibility assessment, and completion of the eCRF. Study data within the eCRF were pseudo-anonymised by SIs at each centre prior to delivery to the contract research organisation for analysis. Data collection was entirely retrospective and did not involve any direct patient contact.

### Study outcomes

Overall survival (OS) was the primary outcome measure and was defined as the time between index date and either date of death or most current medical record at EOFU. This was assessed for both the overall study cohort and stratified by the treatment line in which nivolumab was initiated.

Time on therapy and discontinuation were secondary objectives, where time on LOT was defined from initiation of the treatment to the earliest of the following: date of LOT discontinuation, death, lost to follow-up (LTFU), or the most current medical record at EOFU. Time on LOT was calculated for the overall study cohort, and for subgroups of patients receiving nivolumab at different lines of therapy.

### Treatment switching definitions

In line with previous studies [19] and based on advice from the SIs, treatment switches due to intolerability in this study were limited to 1L treatment only, and applied to switches to/from sunitinib, pazopanib, and tivozanib only as these TKIs share similar efficacy and safety profiles [20]. The implementation of these treatment switching rules ensured the consistent capturing of treatment sequences across all study centres.

Of the original study cohort, six (4.0%) patients were recorded to have received interleukin-2 (IL-2) therapy at 1L, which was not counted as a LOT for the treatment sequencing and treatment pathway analyses.

### Statistical analysis

This study did not test any hypotheses and therefore a formal sample size calculation was not undertaken to determine statistical power. Baseline patient demographic and clinical characteristics were analysed descriptively, where baseline was defined as the index date. Categorical data were reported using frequencies and percentages, with 95% confidence intervals (CIs). Continuous data were reported using mean and standard deviation (SD). Differences between subgroups were assessed using t-tests and chi-squared tests. OS and time on LOT were calculated using Kaplan–Meier methods. Log-rank and Wilcoxon signed-ranked tests were used to assess for differences between subgroups. All analyses were carried out using R version 3.61.

## Results

### Patient characteristics

In total, 151 patients (mean age 66.9 years [SD 10.0], male 72.2% at index), met eligibility criteria, were sampled randomly to be included in the study, and were included in analyses. The median (interquartile range [IQR]) time from aRCC diagnosis to nivolumab initiation was 2.0 years (IQR 0.9–3.6) (Table 1), and median follow-up from index was 15.2 months (IQR 8.7–22.4). Where prognostic risk (International Metastatic RCC Database Consortium [IMDC] classification) was recorded (*n* = 112), 53.5% (*n* = 60) patients were classified as intermediate risk. The majority of patients had an Eastern Cooperative Oncology Group Performance Status (ECOG PS) score of 0 (*n* = 49; 32.5%) or 1 (*n* = 59; 39.1%) at index. The majority of the patient cohort had clear cell histology (*n* = 131; 86.8%) and prior nephrectomy (*n* = 110; 72.8%). The most frequently reported sites of metastasis were lung (*n* = 116; 76.8%), lymph nodes (*n* = 65; 43.0%), bone (*n* = 53; 35.1%), and liver (*n* = 43; 28.5%).

**Table 1:** Patient demographics and clinical characteristics at index (date of nivolumab initiation)

**Variable**	**LOT Nivolumab was received**			**p-value**^b^
	**All patients at index date**^a^ **(*n* = 151)**	**2L** **(*n* = 109)**	**3L + ** **(*n* = 42)**	
***n, % (95% CI) unless otherwise stated***				
***Age (years)***				
Mean (SD)	66.9 (10.0)	67.2 (10.2)	66.2 (9.4)	0.58
Median (IQR)	66.9 (60.4,74.3)	67.4 (59.8,75.3)	66.2 (60.9,70.9)	
***Sex***				
Female	42, 27.8% (21.3,35.4)	28, 25.7% (18.4,34.6)	14, 33.3% (21.0,48.4)	0.46
Male	109, 72.2% (64.6,78.7)	81, 74.3% (65.4,81.6)	28, 66.7% (51.6,79.0)	
***Time from aRCC diagnosis to index date***^a^***(years)***				
Mean (SD)	2.6 (2.5)	2.0 (1.8)	4.2 (3.1)	< 0.01^a^
Median (IQR)	2.0 (0.9,3.6)	1.4 (0.7,2.9)	3.4 (2.0,5.9)	
***ECOG PS at index***^a^				
0 (fully active)	49, 32.5% (25.5,40.3)	38, 34.9% (26.6,44.2)	11, 26.2% (15.3,41.1)	0.66
1 (restrictive)	59, 39.1% (31.7,47.0)	42, 38.5% (29.9,47.9)	17, 40.5% (27.0,55.5)	
2 (off-work)	19, 12.6% (8.2,18.8)	13, 11.9% (7.1,19.3)	6, 14.3% (6.7,27.8)	
3 (limited)	3, 2.0% (0.7,5.7)	3, 2.8% (0.9,7.8)	0, 0.0% (0.0,8.4)	
4 (bedbound)	0, 0.0% (0.0,2.5)	0, 0.0% (0.0,3.4)	0, 0.0% (0.0,8.4)	
Not available^§^	21, 13.9%	13, 12%	8, 19.1%	
***IMDC classification (Heng points) at index***^a^				
Favourable (0)	23, 15.2% (10.4,21.8)	17, 15.6% (10.0,23.6)	6, 14.3% (6.7,27.8)	0.02^a^
Intermediate (1–2)	60, 39.7% (32.3,47.7)	34, 31.2% (23.3,40.4)	26, 61.9% (46.8,75.0)	
Poor (3)	29, 19.2% (13.7,26.2)	25, 22.9% (16.0,31.7)	4, 9.5% (3.8,22.1)	
Unknown	39, 25.8% (19.5,33.4)	33, 30.3% (22.4,39.5)	6, 14.3% (6.7,27.8)	
***Histological subtype***				
Clear Cell	131, 86.8% (80.4,91.3)	93, 85.3% (77.5,90.8)	38, 90.5% (77.9,96.2)	0.39
Non-clear cell: papillary	7, 4.6% (2.3,9.3)	6, 5.5% (2.5,11.5)	1, 2.4% (0.1,12.3)	
Non-clear cell: collecting duct	0, 0.0% (0.0,2.5)	0, 0.0% (0.0,3.4)	0, 0.0% (0.0,8.4)	
Non-clear cell: chromophobe	4, 2.6% (1.0,6.6)	4, 3.7% (1.4,9.1)	0, 0.0% (0.0,8.4)	
Non-clear cell: sarcomatoid	2, 1.3% (0.4,4.7)	2, 1.8% (0.5,6.4)	0, 0.0% (0.0,8.4)	
Not available	7, 4.6%	4, 3.6%	3, 7.1%	
***Prior nephrectomy***				
Yes	110, 72.8% (65.3,79.3)	76, 69.7% (60.5,77.6)	34, 81.0% (66.7,90.0)	0.24
***Site of metastasis***				
Bone	53, 35.1% (27.9,43.0)	37, 33.9% (25.7,43.2)	16, 38.1% (25.0,53.2)	0.77
Brain	19, 12.6% (8.2,18.8)	15, 13.8% (8.5,21.5)	4, 9.5% (3.8,22.1)	0.67
Soft tissue	12, 7.9% (4.6,13.4)	9, 8.3% (4.4,15.0)	3, 7.1% (2.5,19.0)	1.00
Lung	116, 76.8% (69.5,82.8)	85, 78.0% (69.3,84.7)	31, 73.8% (58.9,84.7)	0.74
Lymph nodes	65, 43.0% (35.4,51.0)	47, 43.1% (34.2,52.5)	18, 42.9% (29.1,57.8)	1.00
Liver	43, 28.5% (21.9,36.1)	27, 24.8% (17.6,33.6)	16, 38.1% (25.0,53.2)	0.15
Other	33, 21.9% (16.0,29.1)	20, 18.3% (12.2,26.6)	13, 31.0% (19.1,46.0)	0.14
None	3, 2.0% (0.7,5.7)	3, 2.8% (0.9,7.8)	0, 0.0% (0.0,8.4)	0.66

^a^Index date defined as date of nivolumab initiation; ^b^p-values for tests of differences between treatment subgroups (2L and 3L +); §Due to the re-synthesising of treatment pathways as a result of adjusting for intolerability, some data points, specifically ECOG PS were not collected and therefore have been labelled as information unavailable to differentiate from unknown data points

*aRCC* Advanced renal cell carcinoma, *CI* Confidence interval, *ECOG PS* Eastern cooperative oncology group performance status, *IMDC* International metastatic renal cell carcinoma database consortium, *LOT* Line of therapy, *N* Total population, *n* Sampled population, *SD* Standard deviation

Patient demographics including age, sex distribution, ECOG PS, prior nephrectomy status, and sites of metastases were similar across nivolumab-specific treatment lines (Table 1).

### Transition rates

In total, 72.2% (*n* = 109) of the cohort received nivolumab at second line (2L) and 27.8% (*n* = 42) received nivolumab at third line or later (3L +). Among the 109 patients who received nivolumab at 2L, 52 (47.1%) received further treatment at 3L in the index and follow-up periods; of these, 14 (26.9%) went on to receive fourth line (4L), and of these, 1 (7.1%) went on to receive fifth line (5L). Of the 36 patients that received nivolumab at 3L, 12 (33.3%) received further treatment at 4L and of these, 3 (25.0%) patients received 5L within the follow-up period. Two (40.0%) of the five patients who received nivolumab at 4L transitioned to 5L.

### Time on LOT

**Fig. 1:**
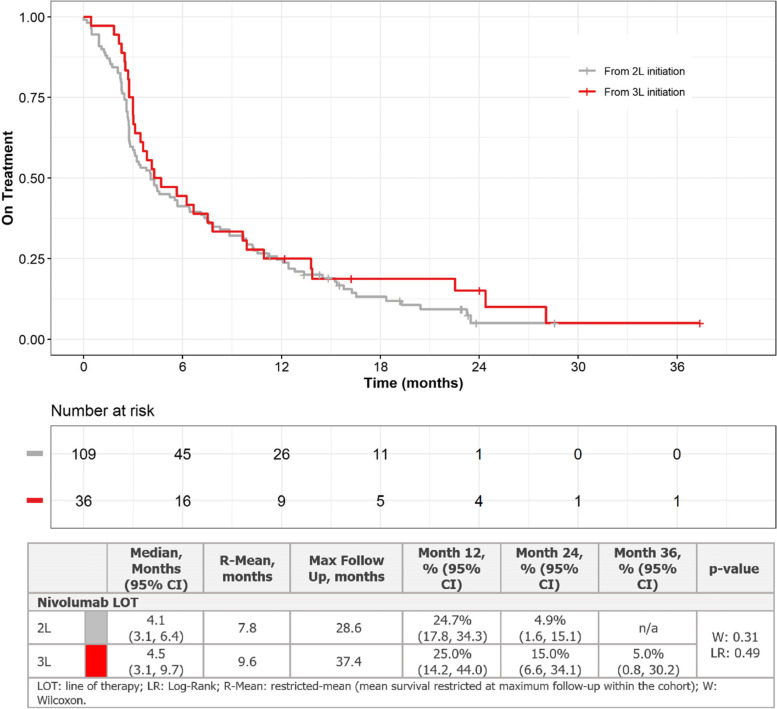
Time on nivolumab LOT

Median time on LOT for any treatment decreased with increasing therapy line, ranging from 7.8 months (95% CI: 6.9, 9.9) in 1L to 4.6 months (95% CI: 3.0, NR) in 5L. For nivolumab-specific LOTs, median time on nivolumab monotherapy was 4.1 (95% CI: 3.1, 6.4), 4.5 (95% CI: 3.1, 9.7), 3.5 (95% CI: 2.1, NR), and 4.3 months (95% CI: NA, NA) for 2L, 3L, 4L, and 5L, respectively (Wilcoxon [W]: 0.41, Log-Rank [LR]: 0.25) (Fig. 1). No patients who received nivolumab at 4L and 5L remained on treatment past 6 months. Percentage of patients remaining on treatment at 12, 24, and 36 months were 24.7%, 4.9%, and NA for 2L nivolumab and 25.0%, 15.0%, and 5.0% for 3L nivolumab, respectively.

### Treatment sequences

**Fig. 2:**
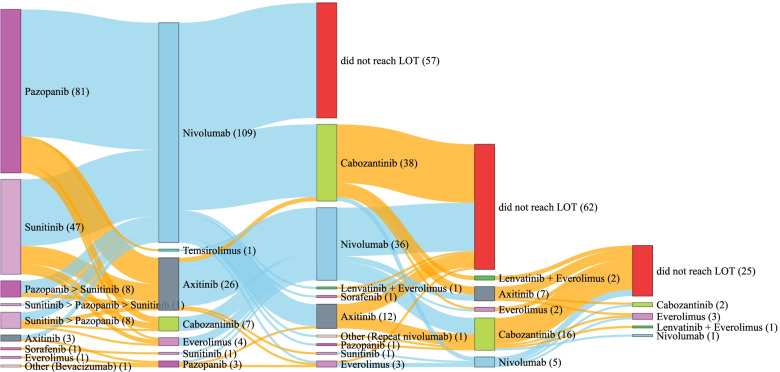
Sankey diagram depicting treatment pathways. Note: > denotes a switch from one treatment to another due to intolerability; + denotes a dual therapy

Pazopanib (*n* = 81; 53.6%) and sunitinib (*n* = 47; 31.1%) were most frequently received at 1L without switching (Fig. 2). First-line TKI switching occurred in 17 (11.3%) patients (pazopanib➔sunitinib, *n* = 8 [47.1%]; sunitinib➔pazopanib, *n* = 8 [47.1%]; and sunitinib➔pazopanib➔sunitinib, *n* = 1 [5.9%]).

All patients who received nivolumab at 2L (*n* = 109) had received TKIs at 1L, including pazopanib (*n* = 63; 57.8%), and sunitinib (*n* = 33; 30.3%). Eleven (10.1%) received both sunitinib and pazopanib at 1L. After receiving nivolumab at 2L, 52 patients (47.7%) went on to receive 3L treatment in the follow-up period, including cabozantinib (*n* = 36; 69.2%), axitinib (*n* = 11; 21.2%), everolimus (*n* = 1; 1.9%), lenvatinib and everolimus (*n* = 1; 1.9%), sofarenib (*n* = 1; 1.9%), or pazopanib (*n* = 1; 1.9%). One patient (1.9%) who received 2L nivolumab also received repeat treatment of nivolumab at 3L (Fig. 2).

At 3L, 36 (38.3%) patients received nivolumab (Fig. 2). Most of these patients had prior treatment with 1L sunitinib or pazopanib and then followed by 2L axitinib (*n* = 22, 61.1%). Five patients received nivolumab at 4L, where prior 3L treatment included cabozantinib (*n* = 2), sunitinib (*n* = 2), and everolimus (*n* = 1).

### Survival outcomes

**Fig. 3:**
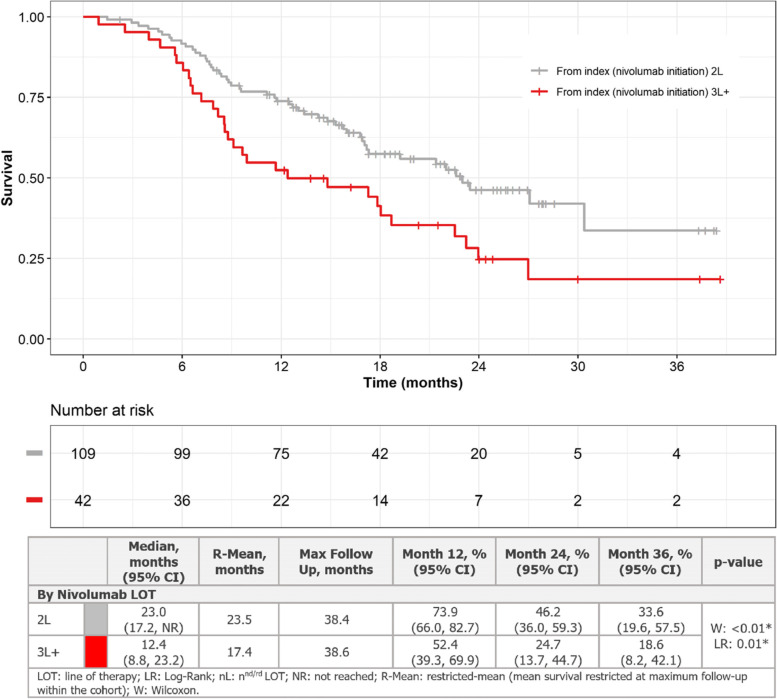
Overall survival for 2L or 3L + nivolumab

Overall, median OS from nivolumab initiation was 19.2 months [95% CI, 16.9–27.0]. Median OS was 23.0 months (95% CI: 17.2, not reached) from initiation of nivolumab monotherapy at 2L, and OS was 73.9% (95% CI: 66.0, 82.7), 46.2% (95% CI: 36.0, 59.3), and 33.6% (95% CI: 19.6, 57.5) at 12, 24, and 36 months, respectively (Fig. 3). In comparison, median OS was 12.4 months (95% CI: 8.8, 23.2) from initiation of nivolumab monotherapy at 3L + , and OS was 52.4% (95% CI: 39.3, 69.9), 24.7% (95% CI: 13.7, 44.7), and 18.6% (95% CI: 8.2, 42.1) at 12, 24, and 36 months, respectively. Similar maximum length of follow-up was observed for 2L and 3L + nivolumab patients (38.4 months and 38.6 months, respectively) (Fig. 3).

### Treatment discontinuation

Of the 151 patients included in analyses, 136 (90.1%) patients discontinued nivolumab monotherapy by the conclusion of the follow-up period (Table 2). The most common reasons for nivolumab discontinuation were progressed disease (including discontinuation due to death) (2L: 61.2%; 3L + : 71.1%) or discontinuation due to adverse events (2L: 34.7%; 3L + : 23.7%).

**Table 2:** Reasons for discontinuation of nivolumab

	**2L** **(*n* = 109)**	**3L** **(*n* = 36)**	**4L** **(*n* = 5)**	**5L** **(*n* = 1)**
Number discontinued	98	32	5	1
**Reason for discontinuation: n, % (95% CI)**				
AE	34, 34.7 (26.0,44.5)	9, 28.1 (15.6,45.4)	0, 0.0 (0.0,43.4)	0, 0.0 (0.0,79.3)
Progressed disease^a^	60, 61.2 (51.3,70.3)	22, 68.8 (51.4,82.0)	4, 80.0 (37.6,96.4)	1, 100.0 (20.7,100.0)
Patient choice	0, 0.0 (0.0,3.8)	1, 3.1 (0.6,15.7)	0, 0.0 (0.0,43.4)	0, 0.0 (0.0,79.3)
Other (please state)	2, 2.0 (0.6,7.1)	0, 0.0 (0.0,10.7)	0, 0.0 (0.0,43.4)	0, 0.0 (0.0,79.3)
Not recorded	2, 2.0 (0.6,7.1)	0, 0.0 (0.0,10.7)	1, 20.0 (3.6,62.4)	0, 0.0 (0.0,79.3)

^a^Including discontinuation due to death

*AE* Adverse event, *CI* Confidence interval, *nL* n^th/rd/st^ line of therapy, *PD* Progressed disease

## Discussion

This study provides insights into the real-world treatment sequences and outcomes of previously treated aRCC patients in clinical practice in the UK who received nivolumab monotherapy. Findings show that nivolumab-specific survival outcomes observed in the real-world setting were comparable to those reported in the pivotal CheckMate 025 trial [15, 16].

The majority of the study cohort were male (72.2%), with a mean age of 66.9 years at index, and had clear cell histology (86.8%). A greater percentage of the study sample had clear cell histology than reported in the wider UK RCC population (86.8% compared with approximately 75%), which may be partially explained by reimbursement instructions requiring a clear cell component or papillary RCC [21], but the remaining patient and clinical characteristics of the study sample were comparable to the broader UK kidney cancer [2, 22] and RCC population [2, 22, 23]. Furthermore, patient characteristics observed in this study were broadly comparable to those reported in CheckMate 025 (median age, 62 years; 77% male) [14].

Treatment sequences observed in this real-world study were largely reflective of NICE recommendations and national treatment guidelines available over the study period [24]. As expected, based on national clinical guidelines [25, 26], patients in this study typically received TKIs either before or after nivolumab monotherapy. For example, pazopanib (64.9%) and sunitinib (42.4%) were the most common 1L treatments. Over two-thirds (67.9%) of 2L nivolumab patients received pazopanib at 1L, either as the only treatment or in a sequence of 1L treatments. At the time of study, recommended 2L and 3L aRCC treatment options in England and Wales included axitinib, nivolumab, cabozantinib, everolimus, or dual therapy lenvatinib with everolimus [17, 27–30]. In our cohort, most patients (61.1%) who received nivolumab at 3L had previously received axitinib at 2L. Cabozantinib most frequently followed 2L and 3L nivolumab monotherapy. However, while NICE recommends everolimus as a 4L therapy option [29], few patients actually received this drug, which may reflect a general trend towards decreased use of single agent everolimus. Patients who reached 4L therapy in our cohort commonly received cabozantinib (50.0%). Among the study cohort, 4 patients across all LOTs received lenvatinib with everolimus; potentially because lenvatinib combined with everolimus is only recommended for those with ECOG PS performance scores of 0–1 and only following one prior VEGF-targeted therapy [24].

Of the 136 patients who discontinued nivolumab regardless of treatment line, 31.6% discontinued due to adverse events.

Median OS (95% CI) for 2L nivolumab patients in this study was 23.0 months (95% CI: 17.22, not reached) and was comparable with median OS reported in the CheckMate 025 trial (25.8 months [95% CI, 22.2–29.8]) [14] and those achieved in similar real-world retrospective studies conducted in Italy (22.5 months) [31], the Netherlands (18.7 months) [32], Croatia, Hungary, and Malta (18.0 months) [33].

There were some methodological limitations in this study. The analysis included results from 151 patients from four sites, which although geographically dispersed NHS England specialist cancer care centres, may not be representative of the wider aRCC population or treatment centres in England and the UK as a whole. Due to the retrospective use of medical record data, results may be affected by incomplete or inaccurate original data and inaccurate data entry using the eCRF, although efforts were made to reduce occurrence of the latter by applying in-built validation checks to the eCRF and the exclusion criterion citing incomplete treatment information. In addition, the representative nature of the study to real world practice may be limited as patients were only eligible for study inclusion if they received nivolumab monotherapy during the index period. As such, the study cohort was more likely to reach EOFU when receiving nivolumab monotherapy as opposed to prior, discontinued therapies. The length of the index period also means that follow-up times varied considerably for the study cohort. This study contained an uneven distribution of patients from each of the centres. Although all centres were part of NHS England, disparity in patient numbers and local practices may affect treatment patterns and outcomes. Finally, it should also be noted that this study is descriptive in nature; and therefore, conclusions on the comparative efficacy between interventions cannot be made.

Despite these limitations, the strengths of this study included the choice of OS as a primary outcome measure, which is considered a robust measure of clinical benefit [34], and less affected by inconsistent recording which limits other intermediate endpoints such as disease progression. The implementation of treatment switching rules also ensured the consistent capturing of treatment sequences across all study centres. Future research could consider related endpoints such as progression-free survival, objective response rate and further investigation into the type and severity of adverse events to support understanding of the clinical effectiveness of treatments in the real-world setting.

## Conclusions

The achievement of comparable OS outcomes for patients who received nivolumab monotherapy in clinical practice compared with clinical trials, as well as adherence to national clinical guidelines, should provide reassurance to clinicians prescribing nivolumab monotherapy, as well as patients receiving treatment. However, descriptions of outcomes based on specific treatment sequences are challenging due to bias that may be introduced through time-varying confounding in comparative analyses of real-world data. Although this study characterises previously treated aRCC patients who went on to receive nivolumab at later therapy lines, additional data from larger cohorts are needed to further assess effectiveness over time in the real-world setting for previously treated aRCC patients.

## Data Availability

The datasets generated and analysed during the current study are not publicly available. Some study data may be available from the study sponsor upon reasonable request. To request study data, please contact R. Carroll (Robert.Carroll@bms.com).

## Abbreviations

1L: First line
2L: Second line
2L +: Second line and beyond
3L: Third line
3L +: Third line and beyond
4L: Fourth line
5L: Fifth line
aRCC: Advanced or metastatic renal cell carcinoma
CIs: Confidence intervals
COVID-19: Coronavirus disease 2019
ECOG PS: Eastern Co-operative Oncology Group Performance Status
eCRF: Electronic case report form
EOFU: End of follow up
HRA: Health Research Authority
ICI: Immune checkpoint inhibitor
IL-2: Interleukin-2
IMDC: International Metastatic renal cell carcinoma Database Consortium
IQR: Interquartile range
LOT: Line of therapy
LTFU: Lost to follow up
mTOR: Mammalian target of rapamycin
NHS: National Health Service
NICE: National Institute for Health and Care Excellence
OS: Overall survival
PD-L1: Programmed death ligand 1
RCT: Randomised controlled trial
RWE: Real-world evidence
SD: Standard deviation
SIs: Study investigators
TKI: Tyrosine kinase inhibitors
UK: United Kingdom
VEGF: Vascular endothelial growth factor

## Glossary

Advanced: Cancer that is unlikely to be curedandgenerally have a poor prognosis
Electronic case report form: Electronic data collection form used to input data by researchers at each centre
Metastatic: A cancer that has spread from its site of origin to different parts of the body
Line of therapy: Each course of treatment is defined as a line of therapy. A patient would move onto the next line of therapy if a treatment has not worked or has been stopped because of the occurrence of side effects or other concerns
Real-world evidence: Evidence or data obtained from real-world practice, which is observational and obtained outside the context of controlled trials
Renal cell carcinoma: The most common type of kidney cancer in adults that originates in the tubules of the kidney
Second line: The second treatment given to a patient once the initial (first-line) therapy has not worked or has been stopped because of the occurrence of side effects or other concerns
Time on line-of-therapy: The time from initiation of a treatment to the end of treatment
